# Fabrication of PLA-Based Nanoneedle Patches Loaded with Transcutol-Modified Chitosan Nanoparticles for the Transdermal Delivery of Levofloxacin

**DOI:** 10.3390/molecules29184289

**Published:** 2024-09-10

**Authors:** Christina Samiotaki, Ioanna Koumentakou, Evi Christodoulou, Nikolaos D. Bikiaris, Marilena Vlachou, Evangelos Karavas, Konstantina Tourlouki, Nikolaos Kehagias, Panagiotis Barmpalexis

**Affiliations:** 1 Laboratory of Polymer Chemistry and Technology, Department of Chemistry, Aristotle University of Thessaloniki, 54124 Thessaloniki, Greece; christinasamiotaki@gmail.com (C.S.); iwanna.koumentakou@gmail.com (I.K.); 2 Laboratory of Pharmaceutical Technology, Division of Pharmaceutical Technology, School of Pharmacy, Faculty of Health Sciences, Aristotle University of Thessaloniki, 54124 Thessaloniki, Greece; nbikiaris@gmail.com; 3 Section of Pharmaceutical Technology, Department of Pharmacy, School of Health Sciences, National and Kapodistrian University of Athens, Panepistimioupoli-Zografou, 15784 Athens, Greece; vlachou@pharm.uoa.gr; 4 Pharmathen S.A., Pharmaceutical Industry, Dervenakion Str. 6, Pallini Attikis, 15351 Athens, Greece; ekaravas@pharmathen.gr; 5 Nanotypos, Stivos, 57020 Thessaloniki, Greece; ktourlouki@nanotypos.com; 6 Institute of Nanoscience and Nanotechnology, NCSR Demokritos, 15341 Paraskevi, Greece; n.kehagias@inn.demokritos.gr

**Keywords:** poly(lactic acid) blends, nanoneedles, nanoencapsulation, drug release, transdermal delivery, levofloxacin

## Abstract

Current transdermal drug delivery technologies, like patches and ointments, effectively deliver low molecular weight drugs through the skin. However, delivering larger, hydrophilic drugs and macromolecules remains a challenge. In the present study, we developed novel transdermal nanoneedle patches containing levofloxacin-loaded modified chitosan nanoparticles. Chitosan was chemically modified with transcutol in three ratios (1/1, 1/2, 1/3, *w*/*w*), and the optimum ratio was used for nanoparticle fabrication via the ionic gelation method. The successful modification was confirmed using ATR-FTIR spectroscopy, while DLS results revealed that only the 1/3 ratio afforded suitably sized particles of 220 nm. After drug encapsulation, the particle size increased to 435 nm, and the final formulations were examined via XRD and an in vitro dissolution test, which suggested that the nanoparticles reach 60% release in a monophasic pattern at 380 h. We then prepared transdermal patches with pyramidal geometry nanoneedles using different poly(lactic acid)/poly(ethylene adipate) (PLA/PEAd) polymer blends of varying ratios, which were characterized in terms of morphology and mechanical compressive strength. The 90/10 blend exhibited the best mechanical properties and was selected for further testing. Ex vivo permeation studies proved that the nanoneedle patches containing drug-loaded nanoparticles achieved the highest levofloxacin permeation (88.1%).

## 1. Introduction

Transdermal drug delivery systems (TDDS) have been extensively explored over recent decades, with the hope of achieving effective drug administration through the skin surface for localized or systemic treatment. It has become a very popular, well-accepted, and promising strategy due to the ease and non-invasive route of administration, the first-pass metabolism avoidance (and the subsequent gastrointestinal side effects that many oral formulations usually entail), the broad flexibility and tunability, and the least discomfort that improves patients’ compliance and prolongs the therapeutic effect [1,2,3]. Since the administered drug is absorbed into the systemic circulation via blood vessels, transdermal delivery can also reduce the fluctuation of drug concentration, providing steady plasma levels with less chances of overdosing [4,5,6]. Finally, transdermal medication offers an increased adaptability in terminating drug administration simply by path removal [7]. Despite the various benefits of the TDDS, this route of administration still does not utilize its full potential due to the inherent barrier of the skin and its physicochemical properties that translate to several obstacles. Only a few therapeutic agents possess the required characteristics to permeate the stratum corneum, located on the epidermis, and to enter blood circulation in sufficient concentrations [8]. Additionally, it is extremely difficult to tune the permeability rate in accordance with individual therapeutic requirements when the delivery of multiple active substances is attempted [5], whereas the possibility of local irritation or contact dermatitis at the site of application, due to the drug or the excipients, still remains a challenge [9].

Recent advances in the field of nanotechnology aim to respond to the aforementioned restrictions. To date, nanoparticle-mediated drug delivery has been a powerful tool, contributing to the development of systems that offer controlled release and facilitate target specificity, thus improving drug efficacy and safety [10,11]. Biodegradable polymeric materials, either synthetic, semi-synthetic, or natural, are very often used for the preparation of nanoparticles (NPs) [12,13]. In particular, the use of natural polymers contributes to their effectiveness, since it demonstrates improved cell proliferation and adhesion, lower toxicity, and enhanced stability in biological fluids, rendering them more biocompatible and less immunogenic [14].

Chitosan (CS) is a natural polymer deriving from chitin and alkaline deacetylation. CS exhibits very low toxicity and good antibacterial properties, and it gradually breaks down to harmless products (aminosugars) that are absorbed from the body. Thus, it is considered an extremely effective drug carrier in biomedical applications [15]. By further modifying chitosan’s structure, tuned properties are enabled (especially with respect to its solubility and swelling capacity), which, in extension, affect the pharmacokinetic profile, as they control the polymer–drug interactions and the loading capability [16,17]. Concerning transdermal delivery, the incorporation of molecules, able to act as permeation enhancers and thus promoting drug penetration, upon the chitosan backbone is a crucial step. Transcutol^®^, or purified diethylene glycol monoethyl ether (DEGEE), is an ethylene oxide derivative with a long history of safe use as a solvent in many products including pharmaceuticals, cosmetics, and food applications [18]. Transcutol currently prefers human and veterinary pharmaceuticals for diverse administration routes, particularly in the context of dermal and transdermal delivery. It provides benefits over alternative enhancers as it is transparent, non-volatile, nearly odorless, and does not compromise the integrity of skin structures. It can promptly penetrate the stratum corneum by modifying the molecular mobility of the stratum corneum protein and lipids, i.e., decreases skin barrier function, and thus alters the skin permeation ability of the active pharmaceutical ingredient. Research encompassing more than 100 active compounds validates its ability to improve drug solubilization, percutaneous absorption rate, and/or retention of drugs in the skin [19].

Yet, despite the significant advantages of NPs, studies report that they alone cannot penetrate beyond the superficial layers of the barrier, and it is highly unlikely, therefore, for the drug to reach the viable cells of the epidermis or beyond when contacting an intact, or partially damaged, skin [20,21]. Nanoneedles then can further and actively enhance skin permeation. Conceptually originated as a miniaturization of microneedles, and broadly defined by high aspect ratio structures with diameter at the nanoscale, nanoneedles constitute an emerging technology for localized, painless, and minimally invasive drug delivery [22,23]. Alike microneedles, these formulations create microscopic pores on the skin, which penetrate the stratum corneum to overcome its barrier properties, thus enabling a wide range of drugs to diffuse into the skin. Nanoneedles are practically invisible and nanometric in size, which offers them the greatest advantage, leading to even more pain-free drug delivery, increasing patient tolerance [24] while enhancing the delivery efficiency by boosting skin permeability [25].

In this work, the chemical modification of chitosan was initially carried out using transcutol in three different ratios (CS/transcutol 1/1, 1/2,1/3) in order to improve the polymer ability to effectively penetrate the lower layers of the skin. The materials were then used to prepare NPs through the ionic gelation method using sodium tripolyphosphate (TPP). The antibiotic Levofloxacin ((*S*)-9-fluoro-2,3-dihydro-3-methyl-10-(4-methylpiperazin-1-yl)-7-oxo-7*H*-pyrido [1,2,3-de]-1,4-benzoxazine-6-carboxylic acid) (Levo) was encapsulated as a model drug in these formulations, and the drug-loaded and neat NPs’ size and morphology were investigated in detail. Next, transdermal patches composed of nanoneedles with pyramidal geometry were prepared using poly(lactic acid) (PLA)/poly(ethylene adipate) (PEAd) blends in various ratios. PLA and PEAd are two promising biobased polymers [26,27], able to form a mostly hydrophobic transdermal patch. The low moisture content in these formulations helps them to remain stable and prevents them from being a completely dried and brittle film, while protecting the material from microbial contamination and limiting the bulkiness of the patches. In addition, studies show that increasing the hydrophobic character of the patch prolongs the regimen of sustained drug delivery through a transdermal route for a period of more than a day [28].

## 2. Results and Discussion

### 2.1. Modified Chitosan Nanoparticles

#### 2.1.1. ATR Spectroscopy

**Figure 1:**
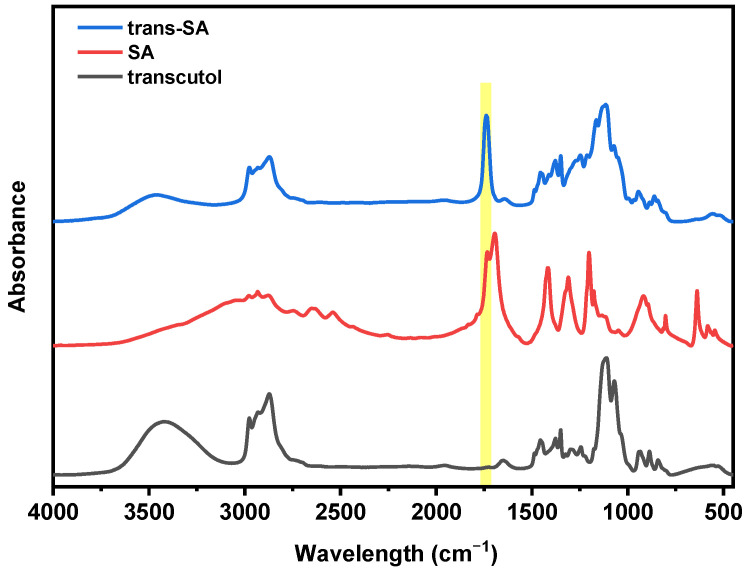
FT-IR spectra of transcutol, succinic anhydride (SA), and the transcutol-SA derivative (trans-SA) (sharp peak at 1720–1690 cm^−1^ with yellow highlight denotes the presence of the carbonyl, C=O).

Figure 1 presents the ATR-FTIR spectra of neat transcutol, succinic anhydride (SA), and modified transcutol (trans-SA). In the transcutol spectrum, a broad peak at 3500–3400 cm^−1^ is observed due to the strain vibrations of the O-H groups. At 2980–2850 cm^−1^, a narrower, multiple-peak band is detected resulting from the vibrational strain of the C-H bonds of the alkyls, while in the fingerprint region, some sharp, low-intensity peaks at 1450–1350 cm^−1^ are distinguished due to the bond vibrations of the -CH_2_ group. At about 1100 cm^−1^, we observe a strong, narrow peak due to the vibrational bending of the C-O bonds found in the ether groups and the alcohol group, while, finally, the small peaks in the 930–880 cm^−1^ region arise from the absorptions of the groups C-C [29]. 

The ATR-FTIR spectrum of SA exhibits a broad peak at about 2900 cm^−1^ resulting from the bending vibration of the alkyl C-H bonds and a sharp peak at 1720–1690 cm^−1^ due to the presence of the carbonyl, C=O. In the fingerprint region, some sharp peaks stand out at 1420–1200 cm^−1^, depicting the absorption bands of the C-O bond, while at 920 cm^−1^, there is another sharp peak, probably due to the bending vibrations of the O-H bond.

From the trans-SA spectrum, the characteristic peak of transcutol at 3500–3400 cm^−1^ is distinguished, while the peaks at 2980–2850 cm^−1^ correspond to the strain vibrations of the alkyl C-H bonds of both compounds. At 1730 cm^−1^, the disappearance of the anhydride double peak can be observed due to the opening of its ring. In addition, a single sharp peak appears at 1730 cm^−1^, which is due to a strong overlap occurring between the absorption band of the carbonyl of the ester bond (1740 cm^−1^) and the carbonyl of the carboxylic acid (1700 cm^−1^) [30]. In addition, the fingerprint region (1450–450 cm^−1^) appears particularly complex with absorption bands due to the stretching vibrations of the C-O and C-C bonds, as well as a variety of bending vibrations. From the above, it is confirmed that the transcutol modification reaction with SA was successful.

**Figure 2:**
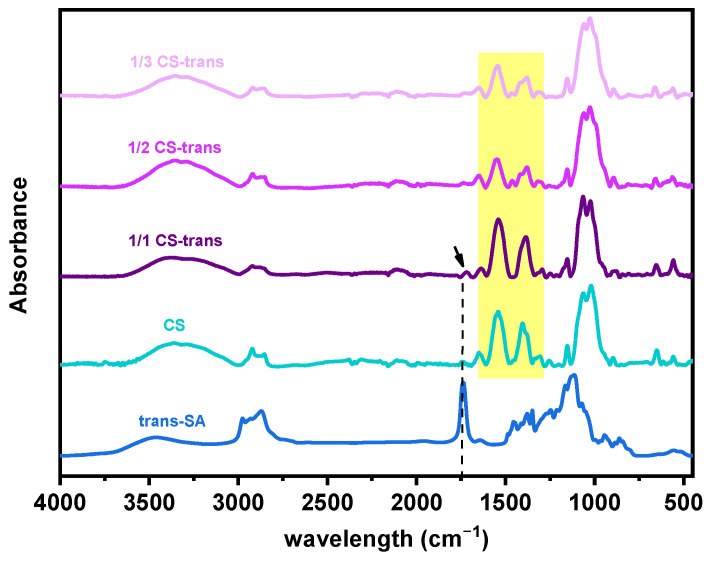
FT-IR spectra of chitosan (CS), trans-SA, and the three different modified chitosan derivatives (CS-trans) at 1/1, 1/2, 1/3 *w*/*w* CS to trans ratio.

After modifying chitosan in the three different ratios and purifying the final samples by Soxhlet extraction, the following spectra were also collected (Figure 2). The typical bands of the CS polysaccharide structure are present at 3400 cm^−1^, due to the O-H vibrations, the stretching vibrations of the N-H bond, as well as the intermolecular hydrogen bonds of the polysaccharide at 1652 cm^−1^ (Amide I), 1570 cm^−1^ (NH_2_ bending) and 1410 cm^−1^ (CH_2_ bending) [31]. Finally, the absorptions at 1080 cm^−1^ and 1027 cm^−1^ are due to the secondary hydroxyl group (C-O bond stress vibration absorption in cyclic alcohols) and the primary hydroxyl group (C-O bond stress vibration absorption in primary alcohols) of the polysaccharide, respectively [32,33].

In the modified CS spectrum, the peak of the carbonyl group at 1730 cm^−1^ is not detected, probably due the reaction held between the C=O groups for the trans-SA and the amino groups of CS. In addition, the peaks at 3400 and 2980–2850 cm^−1^ remain stable, as do the absorptions at 1080 cm^−1^ and 1027 cm^−1^, which come from the CS chain.

#### 2.1.2. Swelling Efficiency

**Figure 3:**
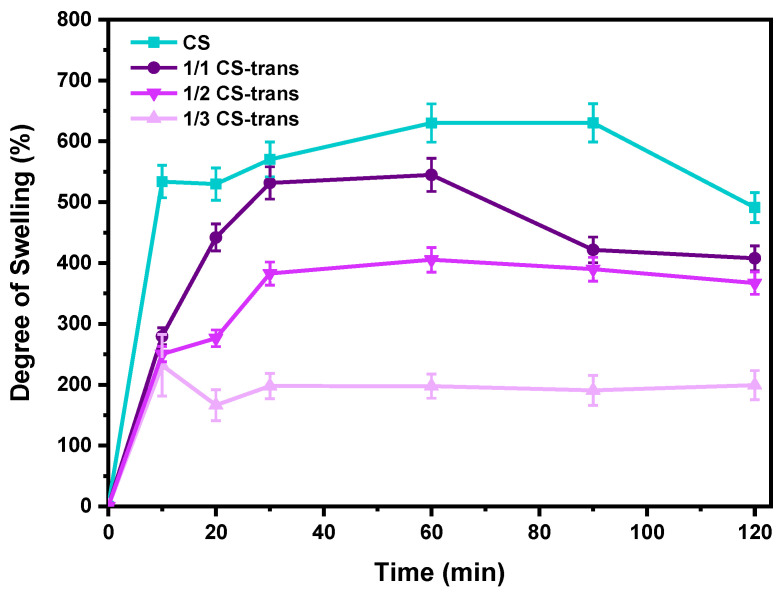
Swelling profile of pristine CS and modified CS-trans in 3 weight ratios for a time period of 120 min in phosphate buffer (pH = 7.4).

Figure 3 presents the results from the swelling experiments. CS reached its maximum swelling degree in the first 60 min (at~630%) and preserved it until 90 min, after which it decreased at~490%. The maximum swelling points of modified chitosan samples occurred at 30 min. The 1/2 and 1/3 samples presented the same pattern and there were no further changes observed after the first 30 min, while the swelling degree of the 1/1 ratio diminished at~421% after 90 min. These results suggest that the modification of CS led to the preparation of new samples with a lower ability to retain moisture in their networks, probably because CS presents more hydrophilic groups than transcutol. In addition, among the modified materials, the swelling degree increased with CS content, a finding that can be attributed to the formation of a compact 3D network after the insertion of transcutol as the polymer side chain.

#### 2.1.3. X-ray Diffraction (XRD)

**Figure 4:**
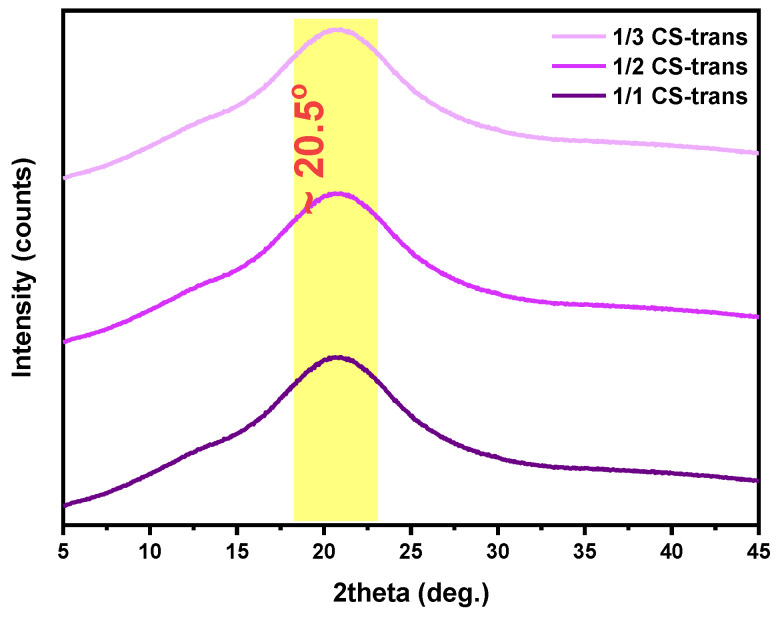
XRD patterns of the three modified chitosan derivatives.

XRD was used to evaluate the crystal structure of the prepared materials. CS is a broadly known semicrystalline polysaccharide [34]. In a series of previous works of ours, we reported its amorphous halo of two characteristic diffraction peaks at 2*θ* = 9–11° and 2*θ* =~22° [35,36,37]. As shown in Figure 4, after modification, a broad peak at 2*θ* =~20.5° was recorded for all three CS-trans derivatives, suggesting a slight increase in polymer crystallinity. This can be attributed to the introduction of transcutol molecules in the polymer chain, which induce stronger intermolecular forces and subsequently lead to a stiffer chitosan backbone with an ordered arrangement that favors the formation of crystals, thus increasing the crystallinity.

**Figure 5:**
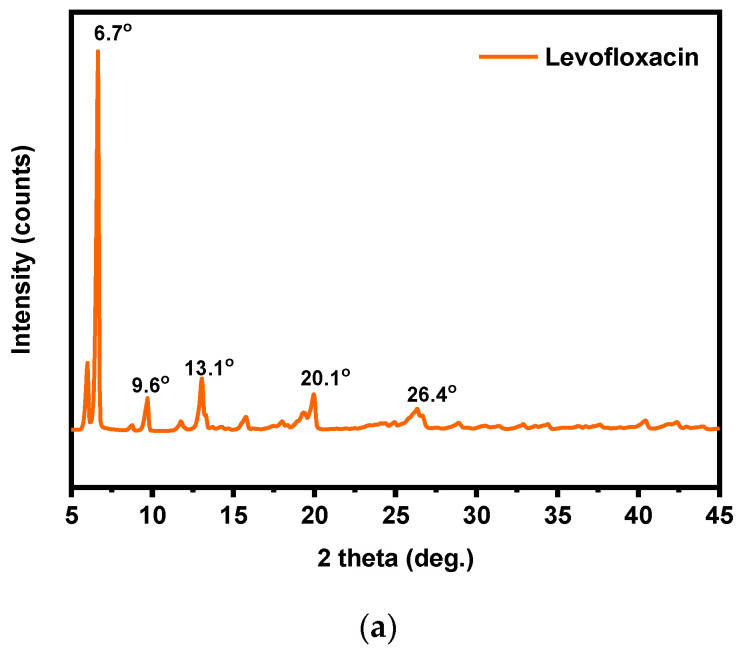

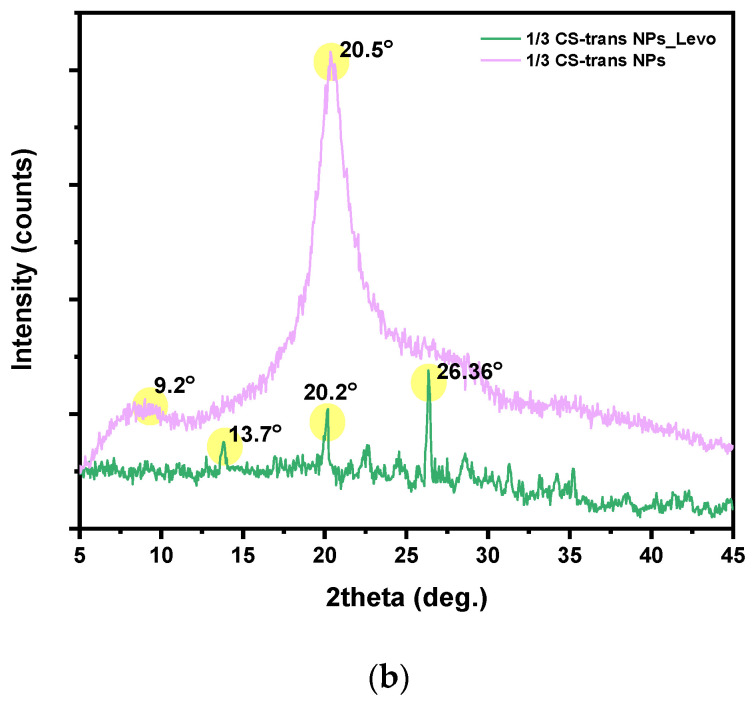
XRD patterns of (**a**) pure Levo drug and (**b**) 1/3 CS/trans NPs and Levo-loaded NPs.

Figure 5 summarizes the XRD diffraction patterns of blank CS-trans NPs, Levo-loaded CS-trans NPs, and the pure drug. The physical state of the drug plays a crucial role in its dissolution rates and consequently its release behavior; thus, the crystalline morphology of the final formulation is a critical parameter to evaluate. As seen (Figure 5a), pure levofloxacin is a highly crystalline compound, recording several characteristic diffraction peaks at 2*θ* = 6.6°, 9.6°, 13.1°, 20.1°, and 26.4° [38]. In the diffractogram of blank modified CS NPs, only the characteristic diffraction peaks of chitosan were detected (9.2 and 20.5°), indicating that the morphology of NPs consists of a dense semicrystalline network of interpenetrating polymer chains crosslinked to each other by TPP counterions. In the case of the Levo-loaded NPs, it is evident that the ionic gelation and the evolved interactions between CS/TPP/drug during nanoencapsulation had a direct impact on the drug’s crystalline structure (Figure 5b). The sharp diffraction peaks at 2*θ* = 6.6 and 9.6° were no longer present in the corresponding XRD pattern, whereas small shifts and declinations in the peak intensity (marked at 2*θ* = 13.7°, 20.2°, and 26.36°) were recorded. These results confirmed that Levo is no longer present as a crystalline compound, but that the largest amount of the drug was encapsulated in the amorphous form inside the NPs [39]. Similar results on the effect of ionic gelation have been previously reported by our group [40,41,42].

#### 2.1.4. Particle Size Determination

As mentioned earlier, in the present study, blank and Levo-loaded NPs were prepared via the ionic gelation method. The particle size and size distribution of NPs are key factors for the development of physically and chemically stable nanocarriers, as they essentially affect the drug release properties and the system’s biological performance.

Following this technique, a series of CS-trans NPs using the three prepared modified derivatives were fabricated according to the method described earlier (Table 1). It was shown that a relatively low particle size was obtained only in the case of the 1/3 CS-trans sample. It should be noted that, although nanotechnology refers to structures having a NP size of up to 100 nm (at least in one dimension), in pharmaceutical applications, it is common to use the term in formulations (or even pure APIs) having particle sizes up to several hundred nanometers, due to their remarkably different properties (as compared to micro-scale formulations) and their unique interactions with the human body [43].

In a further step, following the selection of the most appropriate CS derivate, the effect of the CS-trans/TPP ratio on the size of NPs was also studied (Table 2), and two more weight ratios (1/1 and 4/1) were examined. The findings verified that only the previously tested 2/1 ratio achieved satisfactory results, leading to the formation of NPs with the lowest nanoparticle size. This aligns with previous works of our group where the addition of a higher TPP content caused rapid NP aggregation, resulting in higher particle sizes [44,45].

#### 2.1.5. In Vitro Dissolution of Levofloxacin

Based on the particle size estimation, the final NPs formulation was prepared using the 1/3 CS-trans derivative in a 2/1 CS-trans/TPP ratio. The incorporation of the API into the CS/trans 1/3 system showed a further increase in NPs’ size by ~215 nm, which had, despite this increase, acceptable particle sizes (less than 800 nm) and adequate polydispersity (Table 3). The steric hindrance phenomena induced by the presence of transcutol in the polymer chains results in low drug loading [43].

**Figure 6:**
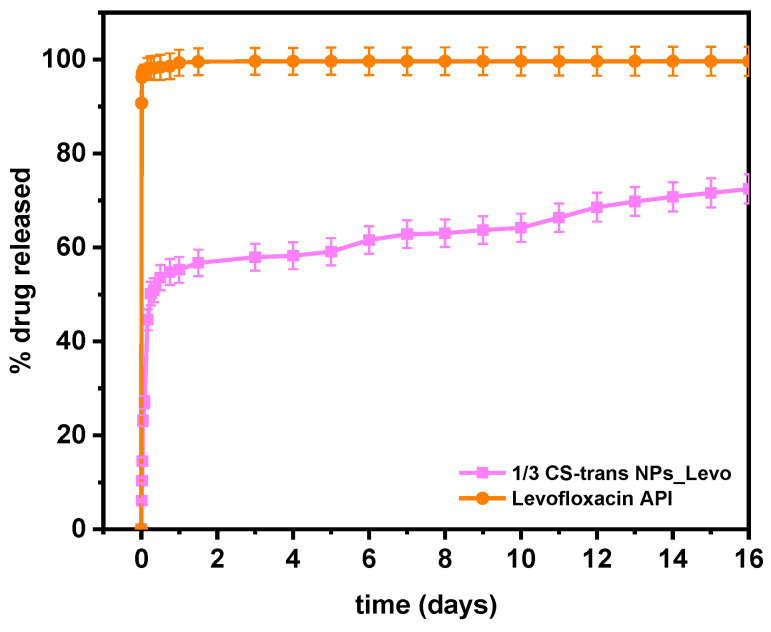
In vitro dissolution profile (%) of pure Levofloxacin and Levo-loaded NPs in 16 days.

Regarding the obtained release profiles (Figure 6), pure Levo, a highly hydrophilic active pharmaceutical ingredient (API), was fully dissolved within the first 5 h, due to its dissociated C_6_-CO_2_H group at pH = 7.4. However, after its nanoencapsulation, we observe a different release behavior. Nanoparticles showed an initial burst release during the first 24 h (~55% of API was released), followed by a slower release rate. By the end of the dissolution experiment (Day 16), the % levofloxacin release reached a maximum value (~72.5%). As can be seen, the burst release is still present, but it was expressed to a much lower extent. This decrease in the dissolution rate is crucial since an immediate high drug dosage can be toxic. The rapid release can be interpreted with regard to the drug deposition on the surface of the nanoparticles, while the slower rates could be associated with the chitosan’s polymer matrix drug release control.

### 2.2. PLA/PEAd Nanoneedles

In a final step, the fabricated NPs were further incorporated in a PLA/PEAd matrix to form the desired nanoneedle-like formulation. The obtained series of samples with varying PLA/PEAd ratios were characterized in terms of morphology and mechanical properties, and the release rates of Levo were also evaluated. The preparation parameters of the S1 to S5 samples are summarized in Table 4.

#### 2.2.1. Nanoneedles’ Morphology

**Figure 7:**
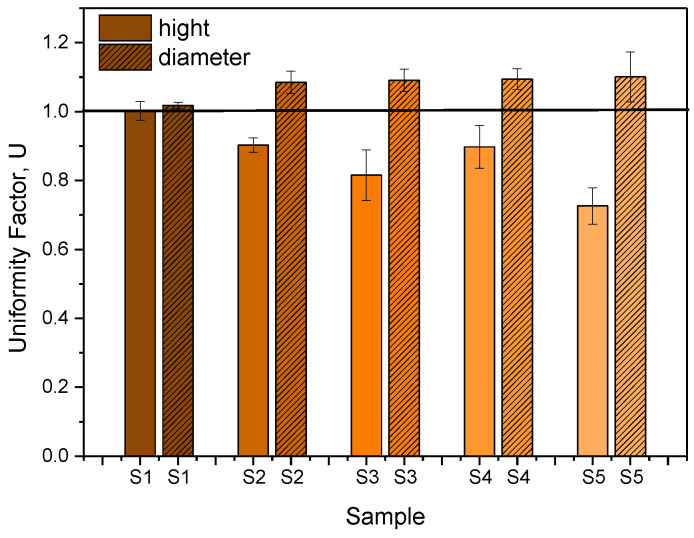
Uniformity factor (U) of the investigated PLA/PEAd nanoneedles.

**Figure 8:**
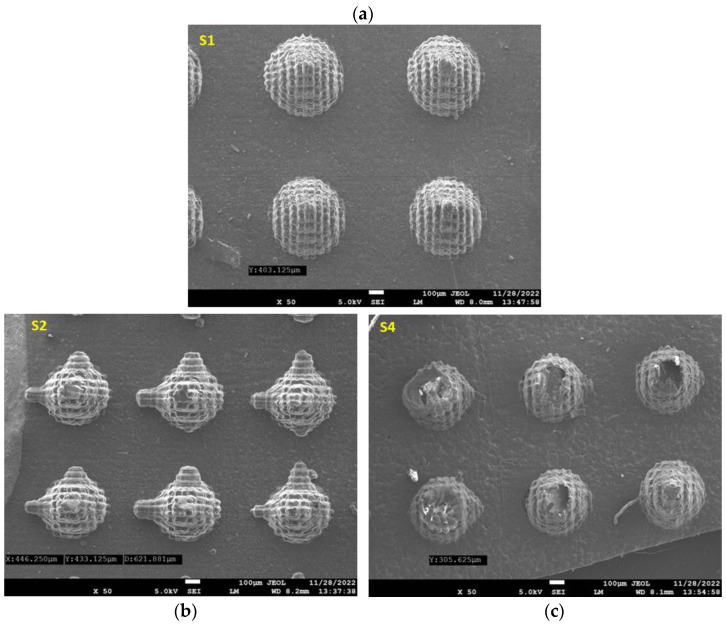
SEM images of the nanoneedles from (**a**) S1, (**b**) S2, and (**c**) S4.

The uniformity factor for all cases was determined in terms of height and base diameter (Figure 7). SEM images (Figure 8) confirmed that the polymeric mixture S1 has dimensions close to the ideal ones, showing uniformly organized needles on the surface of the sample. The needles have several evenly distributed bumps which are due to the way the mold was prepared, while their pyramid shape was verified. However, in the other blends, we observed a fluctuating deviation. These results derive primarily from the mechanical properties displayed by the two polymers. PLA is a hard and durable polymer, which shows very little elasticity [46], while, on the other hand, PEAd is brittle with enhanced elasticity values [47]. Therefore, the decrease in the height of the needles can be attributed to the breaking of their tip during the detachment of the patch from the mold or due to lower mechanical strengths, while, correspondingly, the increase in the diameter of the base may be a result of the greater elasticity of the sample due to the addition of PEAd (Figure 7).

#### 2.2.2. Compression Test

**Figure 9:**
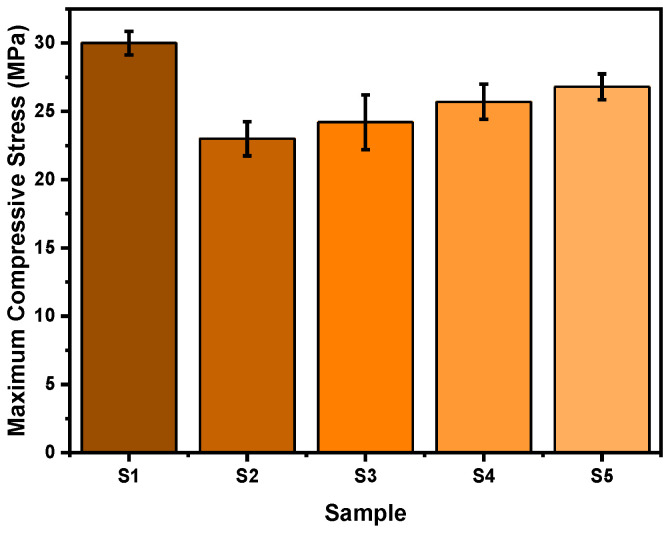
Maximum compressive stress (MPa) of the PLA/PEAd nanoneedles.

In order to evaluate the mechanical properties of the produced microneedles, a compression test was applied to each of the molds. Figure 9 shows the maximum stress value applied to each sample until it was fully compressed and the measurement stopped (flattened). The results revealed that the S1 mixture exhibited the best mechanical behavior since it presented the maximum mechanical stress.

**Figure 10:**
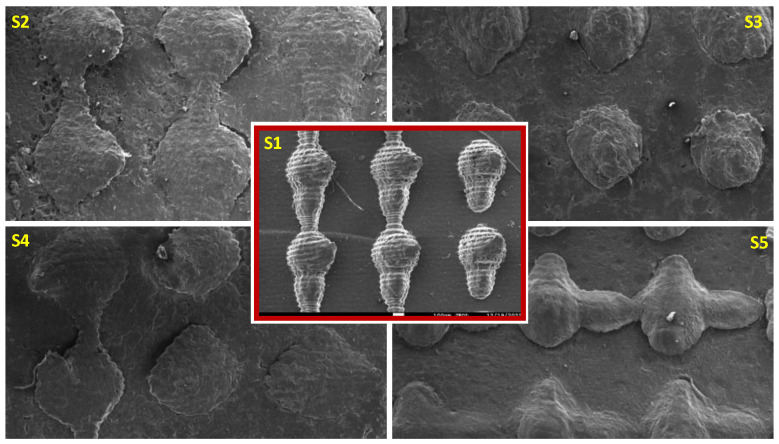
SEM images of the nanoneedles after the compression test.

The above measurements were also confirmed by the SEM images (Figure 10) taken after the compression test. The results revealed that in the S1 mixture, only the pyramid tip was destroyed while the rest of the nanoneedles were completely crushed, suggesting lower mechanical strengths, as predicted before. As a result, S1 was selected for the following ex vivo studies.

#### 2.2.3. Ex Vivo Studies

**Figure 11:**
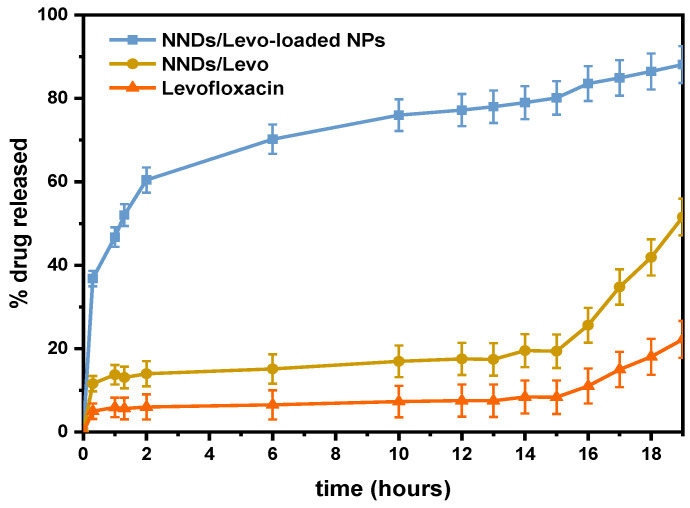
Levofloxacin release profiles from the (blue) PLA/PEAd nanoneedles with CS_Levo NPs, (brown) PLA/PEAd_Levo NNDs, and (red) neat Levo over 20 h of time.

**Figure 12:**
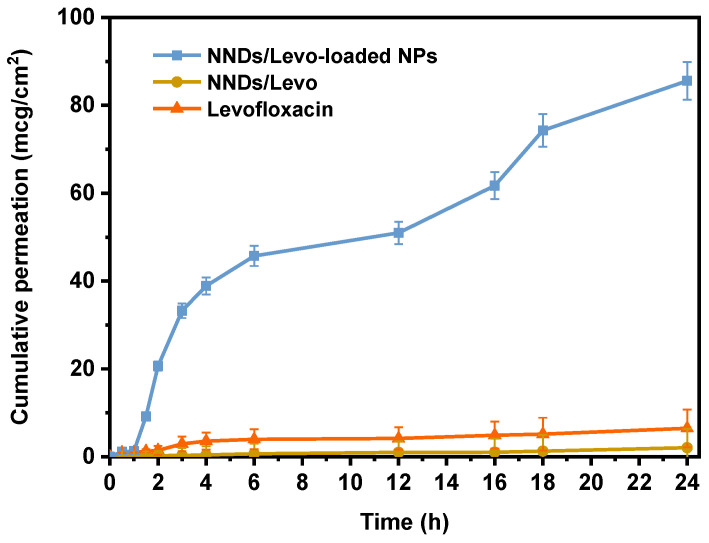
Cumulative permeation (mcg/cm^2^) of levofloxacin from (blue) PLA/PEAd nanoneedles with CS_Levo NPs, (brown) PLA/PEAd_Levo NNDs, and (red) neat Levo through the pork skin over a time period of 25 h.

Ex vivo skin permeation studies are useful for evaluating the amount and rate of penetration of active ingredients. From the graphs below (Figure 11 and Figure 12), it can be concluded that the simply casted patch with no nanostructure embedded on its surface (Levofloxacin) achieves the lowest drug release percentage, ~22.2%, obtained through a biphasic pattern of an initial slow permeation rate up to 15 h, where the drug very slowly penetrates the skin surface (Phase I) and a sharper increment follows, when it is successfully diffused through the model membrane (Phase II). The nanoneedle patch containing pure Levofloxacin in its free form (NNDs/Levo) approaches 51.6%, showing a similar release pattern, but with a substantially enhanced permeability and, in extension, drug bioavailability. Finally, the nanoneedle patch containing Levofloxacin encapsulated in nanoparticles (NNDs/Levo-loaded NPs) performed the highest permeation, reaching a total of 88.1%. The release pattern is again biphasic, but this time, recording a much sharper permeation during the first 30 min, while from that point until 20 h, it shows a continuous permeation of a slow and sustained rate. The above results show that, initially, the nanoneedles managed to penetrate the layers of the skin more effectively than the polymeric patch that did not have the corresponding structure, while the importance of the nanoparticles in the percutaneous penetration of Levo became clear. The results are as expected, given the presence of transcutol, which enhances the penetration ability, but also due to the encapsulation of the active compound in the NP structure which, as reported in the literature, helps the otherwise bulky molecules of levofloxacin reach the circulatory system.

The permeation coefficient values calculated for each formulation are presented in Table 5. The NP-Levo-loaded patch presented the highest drug permeation capability compared with the API and the Levo-loaded nanoneedles.

## 3. Materials and Methods

### 3.1. Materials

CS of high molecular weight (310,000–375,000 Da) and a degree of deacetylation >75% was supplied by Sigma Aldrich Co. (St. Louis, MO, USA). Transcutol (diethylene glycol monoethyl ether, Trans) was supplied by Alfa Aesar (Haverhill, MA, USA). Triphenyl phosphate (TPP) was purchased from Aldrich Chemical Co. (Stainheim, Germany). Levofloxacin (Levo, C_18_H_20_FN_3_O_4_) was kindly donated by Pharmathen Pharmaceutical Company (Athens, Greece). Adipic acid (≥99.0%), ethylene glycol (anhydrous, 99.8%), titanium(IV) butoxide (TBT) (reagent grade, 97%) catalyst, and chloroform (CHCl_3_) (≥99.8%) were purchased from Aldrich Chemical Co. (Stainheim, Germany). Polylactic acid was supplied by NatureWorks LLC (Plymouth, MN, USA), under the trade name PLA 2003D. All other reagents utilized were of analytical or pharmaceutical grade.

### 3.2. Methods

#### 3.2.1. Synthesis of Transcutol-Modified Chitosan

**Scheme 1:**
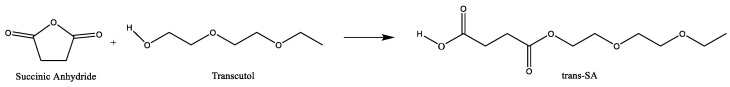
Schematic illustration of the reaction of succinic anhydride with transcutol.

The chemical modification of CS was performed in two steps. First, transcutol (1.8 g) was reacted with succinic anhydride (1.5 g) in a round bottom flask, heated up to 140 °C, and stirred for 2 h to obtain the trans-SA derivative (Scheme 1). Next, the produced residue was dried in an oven at 100 °C for 3 h.

**Scheme 2:**
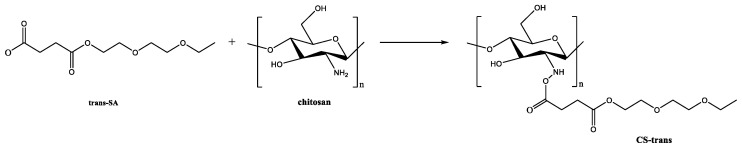
Schematic illustration of the reaction between CS and modified transcutol.

Then, CS (0.5 g) was dissolved in an aqueous acetic acid solution (40 mL, 2% *v*/*v*). The mixture was magnetically stirred at ambient temperature under complete chitosan dissolution. Then, trans-SA (0.5 g) was added to the mixture and left stirring at room temperature for 24 h under continuous magnetic stirring (Scheme 2). The resulting mixture was collected, frozen, and freeze-dried at −80 °C. The desired product with a CS-trans 1/1 ratio was further purified by Soxhlet extraction using ethanol as an eluent in order to remove any unreacted transcutol. The 1/2 and 1/3 samples were prepared in a similar way.

#### 3.2.2. Preparation of Levo-Loaded Nanoparticles

Transcutol-modified CS NPs were prepared according to a previously published modified ionotropic gelation method, using TPP as the ionic crosslinker [42]. Briefly, trans-CS (80 mg) was dissolved in an acetic acid aqueous solution (40 mL, 1% *v*/*v*). An aqueous TPP solution (40 mL, 1 mg/mL) was then added dropwise, and the resultant nanosuspension was left under mild stirring for 1 h. Probe sonication was conducted for 1 min, leading to a better dispersion of the nanoparticles, which were centrifuged (11,500 rpm for 10 min) and lyophilized, using a Scanvac freeze-drier system (Coolsafe 110-4 Pro, Labogen Scandinavia, Kissingen, Germany) for 24 h at −80 °C in order to obtain the final dried NPs in powder form. 

Levofloxacin (Levo)-loaded NPs were prepared accordingly. Levofloxacin (0.04 g) was dissolved in the acidic chitosan solution (2 mg/mL) prior to the addition of TPP, and the procedure was followed as previously mentioned.

#### 3.2.3. Preparation of NP-Loaded Nanoneedles

The synthesis of poly(ethylene adipate) (PEAd) was performed by a typical two-step melt polycondensation reaction, using TBT as a catalyst, as we have previously reported [48], and the PLA/PEAd blends were prepared in five different ratios (Table 5) via solution casting blending using chloroform (CHCl_3_) as the solvent. The preparation processes along with the material characterization are described in our previous work [27]. Then, a 10% *w*/*v* polymer blend solution in CHCl_3_ was poured onto a silicon plate mold of 400 nm (0.4 µm) cavity arrays, supplied by Nanotypos Company (Thessaloniki, Greece). For a more uniform distribution of the polymer blend and better filling into the mold, highg vacuum was applied for 15 min. The samples were left to dry in a fume hood until complete solvent evaporation and the subsequent hardening of the material.

For the incorporation of the drug-loaded NPs into the nanoneedle system, prior to the aforementioned procedure, an appropriate amount of NPs, i.e., 3% (*w*/*w*) NPs/polymer blend ratio, was added to the PLA/PEAd blend solution.

#### 3.2.4. Structure and Properties Investigation

The ATR-FTIR spectra of the samples were recorded using an IRTracer-100 (Shimadzu, Kyoto, Japan) equipped with a QATR™ 10 Single-Reflection ATR Accessory with a Diamond Crystal. The spectra were collected in the range from 450 to 4000 cm^−1^ at a resolution of 2 cm^−1^ (a total of 16 co-added scans), while the baseline was corrected and converted into absorbance mode.

Powder XRD diffractograms were recorded using a MiniFlex 600 XRD system (Rigaku Co., Tokyo, Japan) with CuKa radiation (0.154 nm) over the 2θ range from 5° to 50° with a scanning speed of 1°/min.

Swelling ability was evaluated by measuring the water sorption capacity in phosphate buffer (pH = 7.4). Briefly, CS and CS-trans samples, after being carefully weighed (W_1_), were inserted in water for several hours. At predetermined time intervals (10, 20, 30, 60, 90, and 120 min), the samples were removed, wiped off by filter paper in order to remove the excess surface water, and weighed in order to determine the swelling weight (W_2_). The percentage weight increase in samples during the swelling experiment (i.e., degree of swelling) was calculated by the following equation:

% Swelling Ratio = (W_2_ − W_1_)/W_2_ × 100 (1)

Samples’ morphology was investigated via scanning electron microscopy (SEM). Specifically, the prepared samples were covered with a carbon coating to provide a good conductivity of the electron beam before examining in a JEOL (JMS-840A) scanning microscope (JEOL Ltd., Akishima, Japan). SEM was performed with an accelerating voltage of 20 kV, a probe current of 45 nA, and a counting time of 60 s.

The particle size distribution of the prepared NPs was determined by dynamic light scattering (DLS) utilizing a Zetasizer Nano Instrument (Malvern Instruments, Nano ZS, ZEN3600, Malvern, UK) equipped with a 532 nm laser, using angle measurements of 90° at 25 °C. The samples were measured in suspension form, using an aqueous solution of NaCl (10^−4^ M) after sonication at 25 °C. For all samples, experiments were performed in triplicate, and the results are presented in mean values. Additionally, the particle size and the morphology of the prepared NPs were evaluated via SEM, as described previously.

The determination of % drug loading and % EE was performed by dispersing the prepared nanoparticles (10 mg) in methanol (10 mL). The resulting suspension was stirred for 24 h and filtered using PTFE hydrophobic filters of 0.45 nm in pore size. The Levo content was determined using a Shimadzu HPLC prominence system (Kyoto, Japan), consisting of a degasser (DGU-20A5, Kyoto, Japan), a liquid chromatograph (LC-20 AD, Kyoto, Japan), an autosampler (SIL-20AC, Kyoto, Japan), a UV/Vis detector (SPD-20A, Kyoto, Japan), and a column oven (CTO-20AC, Kyoto, Japan). A CNW Technologies Athena C18, 120 A, 5 μm, 250 mm × 4.6 mm analytical column was used, and the analysis was performed at 25 °C. The mobile phase consisted of methanol/water (acidified with phosphoric acid at final pH = 3.0) 40/60 *v*/*v*, at a flow rate of 1.0 mL/min. UV detection was performed at 295 nm. The injection volume was 10 μL. The calibration curve was created by diluting a stock methanol solution of 500 ppm Levo to concentrations of 0.01, 0.025, 0.05, 0.1, 0.25, 0.5, 1.0, 2.5, 5.0, 10.0, 25.0, and 50.0 ppm [49].

% Drug Loading = (Weight of drug in NPs)/(Weight of NPs) × 100(2)

% EE = (Weight of drug in NPs)/(Weight of NPs) × 100(3)

The in vitro release studies were conducted in a DISTEK Dissolution Apparatus II (North Brunswick, NJ, USA), equipped with an autosampler. Dissolution was performed at 37 ± 0.5 °C, and the rotation speed was set at 50 rpm. The dissolution medium was 500 mL of simulated body fluid (SBF) at pH = 7.4, with 0.1% *v*/*v* of Tween 20 (used to maintain perfect sink conditions). Two milliliters of aqueous solution were withdrawn from the release media at predefined time intervals and quantified via the HPLC method previously described.

Compression tests were performed using an Instron 3340 single column table frame, in accordance with ASTM D695 [50] using a crosshead speed of 1.3 mm/min. Cylinder-shaped specimens (12.7 mm in diameter by 25.4 mm gauge length) were prepared by a supporting jig. At least five measurements were conducted for each sample, and the results were averaged to obtain the mean values of maximum compressive stress. The samples’ morphology before and after the compression test was also investigated via SEM as described above. Their height and diameter values were measured using a microscope (ZEISS SteREO Discovery.V20 Motorized Stereo Microscope, ZEISS, Jena, Germany) and ImageJ (bundled with 64-bit Java 8) to evaluate the uniformity factor, U.

Ex vivo permeation studies were performed on pig ear skin. The ears, received from the abattoir, were cleaned immediately after excision with plenty of running water, hair was removed, and the skin (in 1 mm sample thickness measured with a caliper) was carefully separated from cartilage and the attached fat with a surgical knife. Next, circular skin samples were sectioned, washed with saline, and fixed in the thermostated vertical Franz diffusion cells (PermeGear Inc., Hellertown, PA, USA) between the donor and recipient compartments, with the stratum corneum facing the donor compartment. The diffusion control area has an area of 4.91 cm^2^, and the receiver compartment capacity is 20 mL, following the manufacturer’s specifications. The receiver compartment was filled with degassed phosphate-buffer saline (PBS) at a pH of 7.4 (10 mM), and homogenization was ensured by continuous magnetic stirring (50 RPM), then being thermostated at 37 °C using a hot water circulation system. The skin was initially equilibrated in contact with the release medium for 1 h before the start of the experiment. Films of similar dimensions (2 × 2 × 0.01 cm) and weight (NNDs/Levo-loaded NPs, NNDs/Levo, Levofloxacin) were applied to the skin by wetting with 300 µL buffer solution (for better adhesion) directly to the stratum corneum, and the donor compartment was covered with film (Parafilm M^®^) to avoid evaporation. Permeability was examined for 24 h to avoid the effect of skin degradation. At predetermined time points (0.3, 1, 1.5, 2, 6, 10, 12, 13, 14, 15, 16, 17, 18, and 19 h), samples of 600 µL were taken by needle and syringe from the receiver compartment, with special care to avoid air bubbles. After each sampling, the system was replenished with an equal volume of pre-warmed, degassed buffer to keep the total volume constant. Finally, levofloxacin was quantified (via HPLC) from the fractions collected from the receiver compartment after filtration with a PVDF syringe filter (0.22 µm pore size). The permeation of Levo with time was studied, and the apparent permeation coefficient (P_app_) (cm/h) of the drug was calculated using the following equation:

Papp = ΔQ/(Δt × A × C_0_ × 3600) (4)

J_ss_ = Papp × C_0_ (5)

where ΔQ/Δt is the cumulative permeated amount of drug across the cornea (Q) over time (t), A is the exposed corneal surface area (0.64 cm^2^ in present study), C_0_ is the initial concentration of the drug in the donor chamber, 3600 is the conversion from hours to minutes, and J_ss_ is the steady state transdermal flux (slope of cumulative permeation profiles).

## 4. Conclusions

In the present work, novel transdermal nanoneedle patches were prepared. ATR-FTIR spectroscopy confirmed the successful chemical modification of CS with transcutol in three ratios (1/1, 1/2, 1/3), after which it was used for nanoparticle preparation via the ionic gelation method. DLS results suggest the formation of particles with suitable size (220 nm) only with the 1/3 material, in which Levo was then encapsulated, leading to NPs of 435 nm. From their in vitro dissolution profile, it was concluded that the NPs reach 60% release in a monophasic pattern at t = 380 h. Next, pyramidal geometry nanoneedles were prepared using PLA/PEAd polymer blends in five ratios (90/10, 80/20, 70/30, 60/40, 50/50) and were characterized in terms of their morphology and mechanical compressive strength. S1 (90/10) presented a uniformity factor close to the ideal value, as well as the maximum compressive stress value, suggesting a good mechanical behavior, as proved by the SEM images taken after the compression test, and thus was used for further research. The nanoparticle-loaded polymeric patch was characterized via ex vivo permeation studies which proved that the nanoneedle patches containing loaded nanoparticles showed the highest permeation of levofloxacin (88.1%), almost twice the % release compared to the nanoneedle patch having only the active compound incorporated (not the prepared drug-loaded NPs). This research combines the advantages of biobased and natural polymers along with the state-of-the-art technology of the nanoneedle patch, leading to minimally invasive, painless, and effective drug delivery systems. The observation of levofloxacin high permeation performance, as well as its release profile when incorporated in the nanoparticles, prove the significance of nanotechnology in medical applications and the numerous potentials of such a system in the field of transdermal medication. As future work, we plan to conduct further experiments to assess the permeability and permeation rate of the drug carrier under different temperature and humidity conditions, using appropriate in vitro models to provide a more comprehensive understanding of its stability.

## Figures and Tables

**Table 1:** NPs and their sizes as prepared with the three CS/transcutol ratio estimated by DLS.

NPs Sample Based on Preparation Ratio	Particle Size (nm)	PDI
1/1 CS-trans	620 ± 108	0.642
1/2 CS-trans	390 ± 182	0.591
1/3 CS-trans	220 ± 84	0.345

**Table 2:** NPs size prepared using three CS-trans (1/3)/TPP ratio.

CS-Trans/TPP Weight Ratio	Particle Size (nm)	PDI
1/1	4520 ± 203	0.899
2/1	220 ± 84	0.345
4/1	3840 ± 405	0.563

**Table 3:** NPs and their size prepared with the three CS/transcutol ratio estimated by DLS.

NPs Sample	Particle Size (nm)	PDI	% Drug Loading	% EE
CS/trans-Levo	435 ± 77	0.452	8.97 ± 1.27	24.5 ± 2.38

**Table 4:** PLA/PEAd blends prepared for the nanoneedles patch.

PLA/PEAd *w*/*w* Ratio	Sample
90/10	S1
80/20	S2
70/30	S3
60/40	S4
50/50	S5

**Table 5:** NPs and sizes prepared with the three CS/transcutol ratio estimated by DLS.

Sample	Papp (cm/h)
NNDs/Levo-loaded NPs	0.20791070
NNDs/Levo	1.90731 × 10^−5^
Levofloxacin API	0.00130121

## Data Availability

The original contributions presented in the study are included in the article, further inquiries can be directed to the corresponding author.
